# The saccharibacterium TM7x elicits differential responses across its host range

**DOI:** 10.1038/s41396-020-00736-6

**Published:** 2020-08-24

**Authors:** Daniel R. Utter, Xuesong He, Colleen M. Cavanaugh, Jeffrey S. McLean, Batbileg Bor

**Affiliations:** 1 Department of Organismic and Evolutionary Biology, Harvard University, Cambridge, MA 02138 USA; 2 Department of Microbiology, The Forsyth Institute, Cambridge, MA 02142 USA; 3 Department of Oral Medicine, Infection and Immunity, Harvard School of Dental Medicine, Boston, MA 02115 USA; 4 Department of Periodontics, University of Washington, Seattle, WA 98119 USA

**Keywords:** Symbiosis, Microbial ecology, Pathogenesis, Physiology, Biodiversity

## Abstract

Host range is a fundamental component of symbiotic interactions, yet it remains poorly characterized for the prevalent yet enigmatic subcategory of bacteria/bacteria symbioses. The recently characterized obligate bacterial epibiont *Candidatus* Nanosynbacter lyticus TM7x with its bacterial host *Actinomyces odontolyticus* XH001 offers an ideal system to study such a novel relationship. In this study, the host range of TM7x was investigated by coculturing TM7x with various related *Actinomyces* strains and characterizing their growth dynamics from initial infection through subsequent co-passages. Of the twenty-seven tested *Actinomyces*, thirteen strains, including XH001, could host TM7x, and further classified into “permissive” and “nonpermissive” based on their varying initial responses to TM7x. Ten permissive strains exhibited growth/crash/recovery phases following TM7x infection, with crash timing and extent dependent on initial TM7x dosage. Meanwhile, three nonpermissive strains hosted TM7x without a growth-crash phase despite high TM7x dosage. The physical association of TM7x with all hosts, including nonpermissive strains, was confirmed by microscopy. Comparative genomic analyses revealed distinguishing genomic features between permissive and nonpermissive hosts. Our results expand the concept of host range beyond a binary to a wider spectrum, and the varying susceptibility of *Actinomyces* strains to TM7x underscores how small genetic differences between hosts can underly divergent selective trajectories.

## Introduction

Symbioses in biology exist along a continuum, ranging from facultative, ephemeral interactions to a complete dependence on a host organism [1, 2]. The majority of obligate symbiotic bacteria are known to associate with eukaryotes [3], while far fewer examples of obligate bacteria/bacteria associations have been characterized such as gammaproteobacterial symbionts inside betaproteobacteria [4] and predatory bacteria [5, 6]. Recently, this limited list was expanded with the discovery of *Candidatus* Nanosynbacter lyticus strain TM7x, the first member of the phylum Saccharibacteria to be cultivated. TM7x was co-isolated with a single bacterial host strain and characterized as an obligate epibiotic parasite based on its absolute requirement for host bacteria for propagation and negative impact on the growth of its host under laboratory conditions [7]. The ability of TM7x to grow on a small set of closely-related hosts has been shown previously [7, 8], but the potential range of TM7x hosts remains undefined.

Host selection and specificity are key elements that contribute to parasite impact [9]. Bacterial communities are no exception, affected by obligate parasites such as bacteriophages (phages) and predatory bacteria (e.g., *Bdellovibrio* and *Vampirococcus)*, each with varying ranges of host bacteria [10, 11]. In phages, the better-characterized of the two, host range is defined as the taxonomic diversity of hosts they can infect, and it is most likely confined by multiple factors, including suitable receptors for attachment and the co-option of host machinery for replication [11]. Narrow host range is defined by a phage’s ability to infect only a single host strain or lineage, while phage with broad host ranges can infect multiple strains, potentially spanning several taxonomic levels. Like phage and some predatory bacteria, TM7x are currently considered obligate parasites that complete their lifecycle dependent on a bacterial host [7]. However, the coevolved relationship between the two partners in their natural habitat of the oral cavity may differ, thus warranting a more detailed investigation into the process of host selection and host range.

Saccharibacteria are ultrasmall-sized bacteria with a highly reduced genome compared to typical free-living bacteria, and are placed phylogenetically within the Candidate Phyla Radiation (CPR) super phylum [12–15]. The saccharibacterium *Candidatus* Nanosynbacter lyticus TM7x, HMT-952 (Human Microbial Taxon, HOMD), grew on *Actinomyces odontolyticus* XH001 and its phylogenetically close relatives [7, 8, 16]. Subsequent studies revealed that TM7x induces stress in XH001, and infection of naïve XH001 with TM7x resulted in drastic killing of the bacterial hosts (growth-crash) followed by a recovery phase where host and parasite achieved a long-term stable relationship [8, 17]. Early attempts to characterize TM7x host range used a limited number of hosts, warranting further study [8].

Thus, we mapped the host range of TM7x more fully and explored the physiological and genomic features associated with the various phenotypes. Beyond their ability to support TM7x growth, TM7x hosts showed a wide range of initial responses to TM7x infection. Furthermore, these differential phenotypic responses were reflected in the gene content of the host bacteria. To the best of our knowledge, we present the first thorough analysis of saccharibacterial host range and their associated phenotypes.

## Materials and methods

### Bacterial strains and culture conditions

Bacterial strains, their sources, and growth conditions are listed in Table S1. Before each experiment, cells from frozen stock were recovered and passaged twice in BHI to ensure homogeneity. Most oral *Actinomyces* spp. are facultative anaerobes, but some are anaerobic while others are aerobic. We used microaerophilic conditions due to our previous finding that XH001 and TM7x grow best in microaerophilic conditions [17].

### Host range and re-infection assay

TM7x cells were isolated away from their initial bacterial host (XH001) using a previously developed method [8]. To infect new *Actinomyces* and other oral bacterial strains, bacterial hosts were pelleted and resuspended in 4 mL fresh media (Table S1). To this, TM7x cell suspension was added. Cocultures were incubated microaerophilically (2% O_2_, 5% CO_2_, 93% N_2_) in a Whitley workstation at 37 °C for 24 h before passaging.

**Fig. 1:**
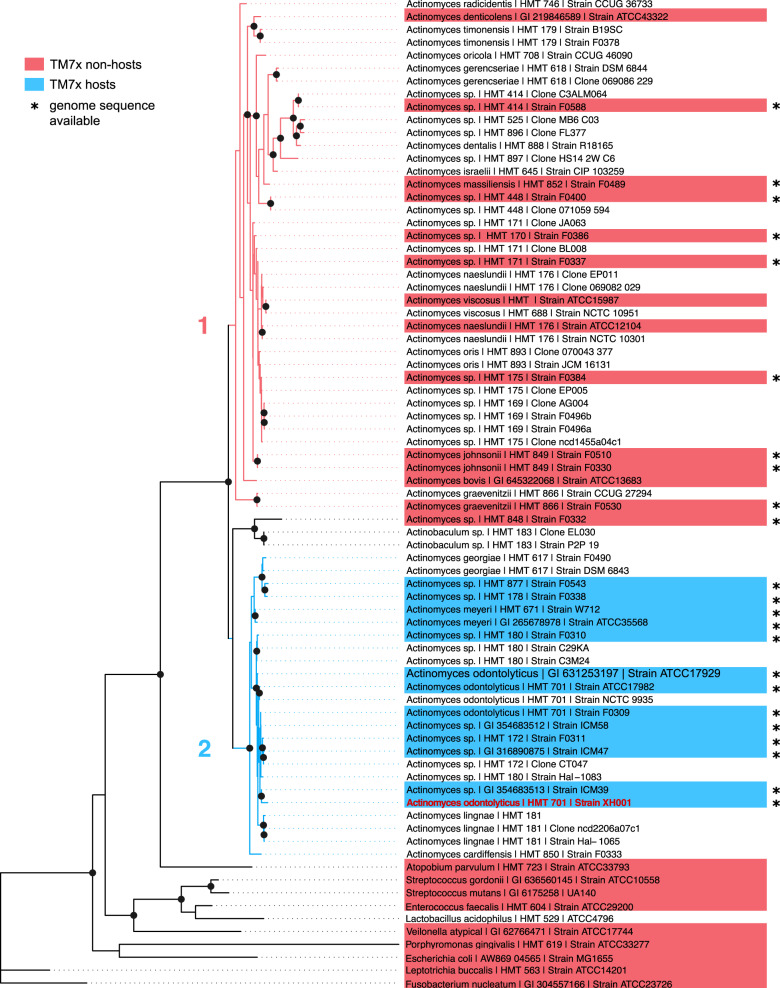
TM7x host-range. Different *Actinomyces* and oral bacterial species (highlighted) were tested for TM7x re-infection. A maximum-likelihood phylogenetic tree was created using the 16S rRNA gene sequences of candidate hosts, which revealed two *Actinomyces* clades (1 and 2). XH001 (orange) is the host with which TM7x was originally isolated. Susceptible and resistant strains are shown in blue and red, respectively. Bacteria included for phylogenetic analysis but not tested for TM7x susceptibility are not highlighted. The 23 strains indicated by asterisks have sequenced genomes publicly available. The scale is 4 substitutions per site. Nodes with bootstrap support ≥70 are marked with a black dot.

Thirty-seven candidate hosts were infected and passaged every 24 h into fresh BHI media at a 1:10 dilution (Fig. S1). The passaging was designed to mimic continuous culture as much as possible to establish that nutrient limitation was not a factor for growth. Host strains were selected based on their phylogenetic diversity and strain availability from the collection centers (Fig. 1). After 5–8 passages, infected cocultures were tested for TM7x presence by PCR, phase-contrast imaging, and fluorescence in situ hybridization (FISH) using Saccharibacteria-specific probes [8] (see supplementary information).

To determine growth-crash and TM7x score in susceptible strains, the infection of 12 strains was carried out as described above. Subsequently, at every passage, optical (cell) density at 600 nm (OD600) was measured using a Spectronic Genesys 5 spectrophotometer, and TM7x score was determined by phase-contrast microscopy to score the amount of TM7x [8] from 0 to 1 for no to high amount of TM7x bacteria (Fig. S2). This qualitative scoring method considered both free-floating and host-attached TM7x cells. This method was validated previously by qPCR using Saccharibacteria-specific primers [8]. Cell lengths of all susceptible *Actinomyces* were analyzed according to previous methods [17] (see supplementary information, Fig. S3).

### Re-infection assay with TM7x gradient

Isolated TM7x cells were quantified by a previously described method [8]. Briefly, TM7x cells were diluted, filtered onto a 30 nm PCTE filter (SterliTech) membrane, and labeled with 1:400 diluted SYBR Gold (Invitrogen) solution. Cells were numerated in each field of view and back calculated to determine the TM7x cells per milliliter.

Since TM7x isolation and quantification require extensive time and effort, we prepared TM7x cells separately for each XH001, W712, and ICM47 host experiments (Figs. S4, S5, S6). Hence, the ratio added to each experiment differed. To set up the experiment, we pelleted replicate tubes of 400 μL of 0.2 OD hosts and resuspended them in 200 μL of BHI. To each tube, a different ratio of TM7x was added and diluted to a final volume of 2.5 mL. Besides TM7x score and OD600 monitoring, we also plated these cocultures on 5% sheep blood agar plate at each passage to determine the total colony forming units and irregular colony numbers [8]. Cultures were plated in triplicate, and the graphs report the mean and standard deviation as error bars. We FISH imaged each host during the re-infection experiment (Fig. S7).

### 16S rRNA phylogenetic analysis

Full-length 16S sequences were obtained from public databases, primarily HOMD and NCBI, and used to generate a maximum-likelihood tree with RAxML [18] using a GTR + GAMMA model for 1000 bootstrap generations on the pairwise alignment.

### Pangenome generation and comparative genome analysis

A narrative methods document providing a reproducible workflow for all genomic analyses can be found on Dr Bor’s lab website at https://www.forsyth.org/labs/bor-lab/. Of all tested strains, 23 *Actinomyces* strains had publicly available genomes which were downloaded from NCBI. Amino acid identity (AAI) was calculated for all genome pairs with CompareM (https://github.com/dparks1134/CompareM; S8a). Briefly, this method calls genes using Prodigal [19] and subsequently computes amino acid similarity between gene pairs with DIAMOND [20], resulting in the heatmap Fig. S8a, from which the *A. odontolyticus* XH001 row was extracted and added to the pangenome. A phylogenomic tree (Fig. S8b) was also generated with PhyloPhlAn2 [21], following developers’ recommendations. Briefly, we created a reference set of core genes based on *A. odontolyticus* and subsequently restricted PhyloPhlAn2 to use only orthologs found in all 23 genomes and a diversity estimate of ‘medium’. PhyloPhlAn2 identifies single-copy genes present in each genome, extracts the most informative subsequences of each gene, concatenates them, and generates a consensus maximum-likelihood phylogenetic tree [21]. Trees were visualized in R with the ape and phytools packages [22, 23].

The pangenome (10.6084/m9.figshare.12410360) was created from the genome sequences using anvi’o, following a standard pangenome workflow [24]. Briefly, the program anvi-gen-contigs-database called genes using Prodigal [19] and used anvi-run-hmms with a hidden Markov model [25] to evaluate genome completeness and redundancy based on the fraction of single-copy core genes found or duplicated, respectively. Gene sequences were associated into gene clusters, operationally defined groups of putatively homologous genes, using MCL [26] on the amino acid similarities through the anvi-pan-genome program with BLAST [27].

Functional enrichment analyses among the groups defined by TM7x susceptibility were carried out using the program anvi-get-enriched-functions-per-pan-group with default parameters [28]. This program scores enrichment by comparing the observed proportion of each function among genomes split according to TM7x susceptibility (resistant, permissive, and nonpermissive).

Phylogenetic relationships were constructed for all gene clusters core to all 23 genomes and the 13 susceptible genomes (selections “All core” and “Susceptible core” in the pangenome). FastTree [29] calculated an approximately maximum-likelihood phylogenetic tree from each gene cluster using default parameters. The resultant trees were then screened using a custom Python script (included in the reproducible methods) that identified gene clusters matching one of the three specified topologies. The associated functional prediction for each of these gene clusters were retrieved from the master gene cluster table (Table S2).

## Results

### TM7x has restricted host range

In this study we expanded the number of *Actinomyces* host species/strains that were previously tested on TM7x infection [8] and conducted thorough phenotypic and comparative genomic analyses. TM7x cells were isolated apart from their original co-cultivated bacterial host XH001 (*Actinomyces odontolyticus* strain) and added back to cultures of diverse *Actinomyces* strains (*n* = 27) that span the *Actinomyces* lineage, as well as other common oral bacterial strains (*n* = 10) in an established re-infection assay (see methods, Table S1). By 16S phylogeny, *Actinomyces* lineages are divided into two major clades (clade-1 and −2), with XH001 in clade-2, agreeing with previous study [8] (Fig. 1). TM7x did not grow on any clade-1 *Actinomyces* strains after multiple passages, nor the common oral bacteria; while all tested strains (12 in addition to XH001) in clade-2 were infected with TM7x over multiple passages (Fig. 1) based on imaging techniques and PCR. These results suggest that the tested *Actinomyces* species fall into two major groups: resistant or susceptible to TM7x infection (Fig. S1a).

### Different phenotypic responses of bacterial hosts to TM7x infection

Infection of naïve XH001 cells by TM7x induces a “growth-crash”, in which host cell density drops precipitously, followed by recovery in their bacterial hosts (Fig. S1b) [8]. This is analogous to a previously hypothesized cyclically-recurring population crash during parasite-host dynamics [30, 31], but interestingly in our case only a single crash was observed followed by stable growth. Recovered XH001 were found to have single-nucleotide variants relative to their naïve ancestors, presumably imparting the observed regain of fitness [7, 8]. The host growth is measured by cell density (OD600) whereas TM7x abundance is scored visually by phase-contrast imaging (see methods; [8]. These methods assess the host and TM7x abundances qualitatively but rapidly and accurately [8].

**Fig. 2:**
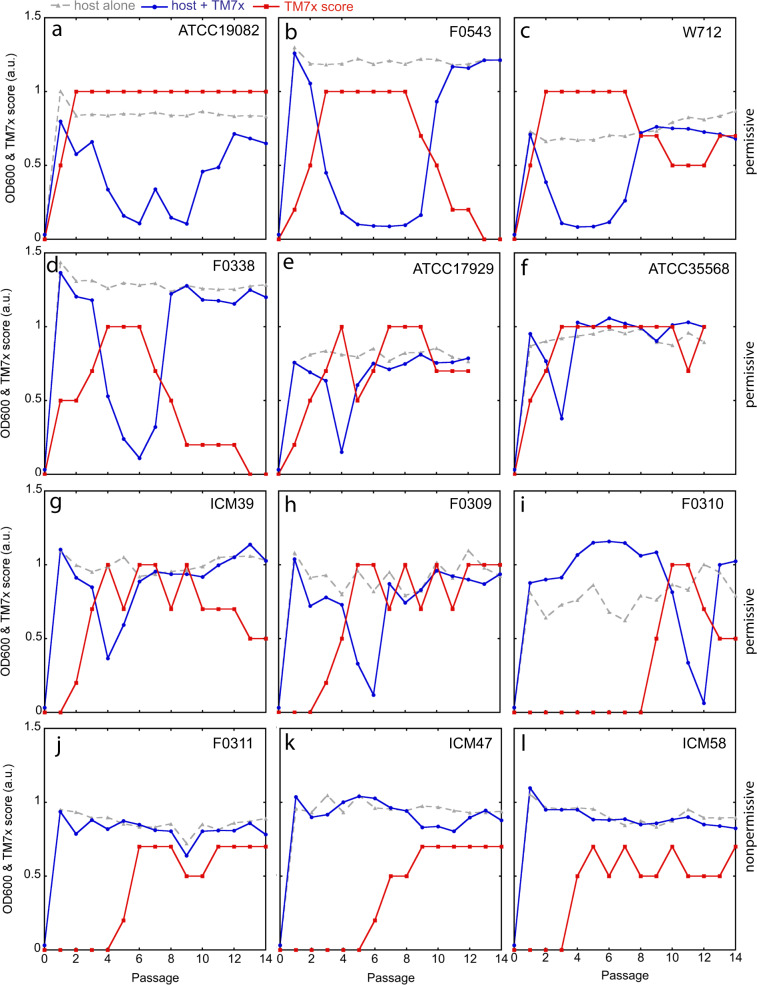
Re-infection of susceptible bacteria by TM7x. **a**–**l** Isolated TM7x cells from XH001-TM7x coculture were added to the 12 susceptible host cells at passage 0, and cell density (blue, circles) and TM7x scores (red, squares) were monitored during subsequent passages. Host alone control is shown in gray triangles. **a**–**i** Host strains where cell density drops precipitously are referred to as ‘permissive’ hosts. **j**–**l** Three strains that do not have growth-crash are termed ‘nonpermissive’ hosts. Host strain names are labeled on the top right corner of each graph.

To further investigate the initial response to TM7x infection, the re-infection assay was conducted by adding TM7x to the 12 susceptible *Actinomyces* strains with a three-to-one TM7x-to-host cell ratio, and their growth was monitored by OD600 and TM7x scores (Fig. 2). Nine of these hosts displayed varying growth/crash/recovery patterns, and all of these included a clear crash phase and thus are referred to as “permissive” hosts (Fig. 2a–i). However, the remaining three hosts (F0311, ICM47, ICM58) lacked a discernable crash phase, hereafter referred to as “nonpermissive” hosts (Figs. 2j–l, S1c). Furthermore, three of the nine permissive hosts (ATCC17982, F0543, W712) had extended, 4–5 passage-long growth-crash phases before recovery while the rest of the hosts had only one passage-long growth-crashes (Fig. 2a–c). TM7x scoring was consistent with the observed host growth-crashes. When initial increase of the TM7x score was plotted for all hosts (Figs. 2, S1d), the three nonpermissive hosts (F0311, ICM47, ICM58) had a late increase in TM7x score compared to the rest of the hosts. F0310 was the only permissive host to have very late TM7x increase and growth-crash at passage twelve (Fig. 2i).

Previously, during the growth-crash phase, both attached and free-floating TM7x cells were observed, with individual XH001 cells often infected with multiple TM7x cells [8]. This induced host cell swelling and elongation, both common morphological stress responses, with XH001 cell length increasing from ~1.7 µm in monoculture to ~3.7 µm in cocultures, and eventually led to cell death [17]. Phase-contrast imaging illustrated similar results, with increased numbers of attached and free-floating TM7x observed for all nine permissive hosts and one nonpermissive host (Fig. S2a–i, j). However, two of the nonpermissive hosts (ICM47, IMC58) did not display an increased level of TM7x bacteria on their surfaces, nor increased cell length (Fig. S2k, l). To assess cell length quantitatively, we measured the cell length for all 12 bacterial hosts after infection. All hosts had significantly increased cell length (Figs. S1e, S3a, b, d–j) except two nonpermissive (ICM47, ICM58) and one permissive (W712) strains maintained or even slightly decreased their cell length after TM7x infection (Fig. S3c, k, l). The decrease in W712 cell length could be a result of W712 having the longest cells before TM7x infection or an inherent limitation in the image analysis of long cells (see methods). Nevertheless, W712 cells were swollen when they were infected with TM7x (Fig. S2c). Furthermore, although F0311 is a nonpermissive host, it did show many TM7x bacteria on its surface during the infection (Fig. S2j), which could be contributing to its increased cell length. Our findings suggest that TM7x-susceptible hosts divide into two broad categories (Fig. S1a): permissive and nonpermissive, though the permissive strains do present a spectrum of crash intensity and duration (Fig. 2).

### Host sensitivity to TM7x infection

**Fig. 3:**
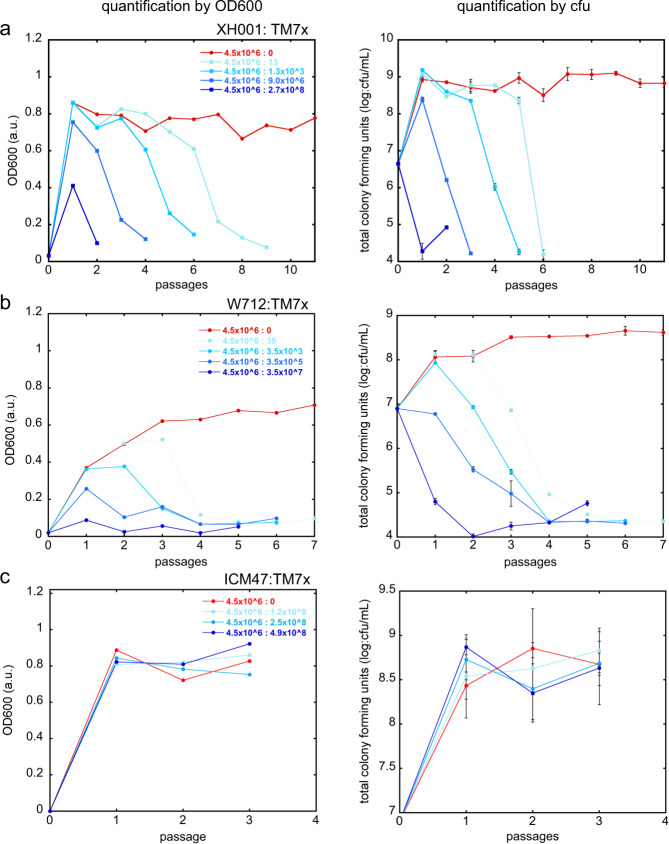
Host sensitivity determined by varying TM7x dosage. Isolated TM7x cells were added to host cells XH001 **a**, W712 **b** and ICM47 **c** in increasing concentrations. For each concentration of TM7x, shown as a TM7x to XH001 ratio, cell density (column one) and total colony forming units (column two) were determined, and only the region leading up to the growth-crash point is graphed. The full data are shown in Figs. S4–6. Total colony forming units were determined in triplicate and error bars indicate the standard deviation.

Our data showed that even though similar TM7x-to-host ratios were used in re-infection experiments, different hosts displayed drastically different crash/recovery dynamics (Fig. 2), suggesting these hosts have differential sensitivity to TM7x. Notably, a rapid increase of TM7x abundance within the first two passages was observed for three strains: *A. odontolyticus* ATCC17982 and two *A. meyeri* strains (W712 and ATCC35568) (Fig. 2a, c, f). To investigate this differential sensitivity further, dose-dependent TM7x infection of naïve XH001 cells was carried out. Results showed that the passage at which XH001 crashed, referred to as the ‘crash point’, was TM7x concentration dependent —with increasing TM7x, we observed earlier crash points (Figs. 3a, S4). Total colony forming units and irregular colony numbers, reflecting the number of total viable hosts and the TM7x infected hosts, respectively [8], were also determined during all passages. By these measurements, the crash points were dependent on the number of TM7x added to the assay. TM7x was able to infect at extremely low concentrations (three TM7x per 4.5 × 10^6^ XH001 cells), and able to completely inhibit XH001 at higher concentrations (2.7 × 10^8^ TM7x per 4.5 × 10^6^ XH001 cells). A similar pattern of TM7x and XH001 growth dynamics were observed at each TM7x concentration (Fig. S4). During the XH001 crash phase (by OD600 or total cfu), the amount of TM7x (by TM7x score or irregular colony) always increased to a maximum and then decreased during XH001 recovery. The crash points determined by total colony forming unit always occurred ~1–1.5 passages before the OD600 crash point, which was consistent with our previous study [8]. This passage difference may be explained by the fact that dead cells can contribute to the cell density measurements.

The sensitivity of *A. meyeri* strain W712 to TM7x was similarly tested. Remarkably, while dose-dependent growth-crash was also observed (Figs. 3b, S5), it took close to tenfold fewer TM7x cells (3.5 × 10^7^ TM7x per 4.5 × 10^6^ W712 cells) to completely inhibit the initial growth of W712 compared to XH001 (Fig. 3b), suggesting that the sensitivity of W712 to TM7x allows faster TM7x growth at the expense of W712. This was reflected by both the OD600/TM7x score and total/irregular colony measurements (Fig. S5). Again, similar to what was observed in the initial coculture experiment (Fig. 2c), all growth-crashes in W712 had prolonged growth-crashes (Fig. S5). In contrast, the nonpermissive strain ICM47 was completely resistant to growth-crash even at the TM7x-to-ICM47 ratio of 4.9 × 10^8^:4.5 × 10^6^ (Figs. 3c, S6). Despite TM7x infection and growth on ICM47, no growth-crash was observed by cell density measurement and total colony forming units. ICM47 strains also did not form obvious irregular colony morphology, suggesting TM7x does not stress or damage host growth as with the other strains.

### TM7x has unique cell localization on the nonpermissive ICM58

**Fig. 4:**
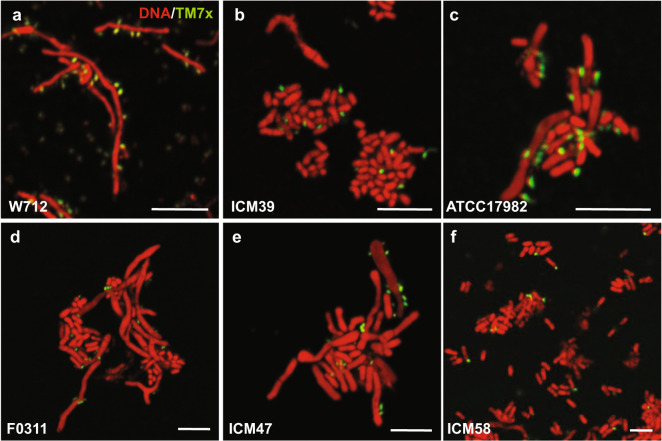
TM7x localization on ICM58. FISH imaging was carried out for all permissive (**a**–**c**, see Fig. S7) and all nonpermissive (**d**–**f**) bacterial hosts. TM7x (green) was visualized using a Saccharibacteria-specific DNA probe tagged with the Cy5 fluorescent molecule. The host bacteria were visualized by universal nucleic acid stain syto9, which also stains TM7x. Only sample strains are shown in this figure, and the complete set can be found in Fig. S7, including a few of the resistant strains visualized by FISH. Scale bars are 5 μm.

TM7x and XH001 have various morphological cell shapes depending on growth conditions and nutrient availability (Fig. S7a) [17]. For all permissive and nonpermissive strains, we observed normally shaped TM7x bacteria growing on the cell surface of the host bacteria by FISH (Figs. 4, S7). Consistent with our previous findings, TM7x attached to bacterial hosts had simple dot/cocci or teardrop-like morphology, shown in green (Figs. 4, S7) [17]. Remarkably, compared to all tested bacterial hosts, only on ICM58, many TM7x localized to the cell poles (Fig. 4f). The polar localization was previously not observed in the close relatives of TM7x, but was shown in a distant lineage (HMT-351) that grows on *Actinomyces* sp. HMT-897 [32]. Exactly how and why pole localization occurs is yet to be determined. Typically, gram-positive bacteria have significant long-axis polarization in terms of protein composition and cell wall structure [33], and TM7x could be targeting those areas. The polar localization of TM7x on ICM58 suggests a different mechanism for attachment compared to other hosts.

### Genome content separates permissive and nonpermissive hosts

As genomes of twenty-three out of the twenty-seven tested *Actinomyces* strains are publicly available, we downloaded them for comparative genomic analyses. To place the currently unnamed genomes (e.g., *Actinomyces* sp. F0310) in context with named species, we first related genomes by average AAI and constructed a phylogenomic tree from concatenated core genes (Fig. S8a, b). From the AAI data, clear patterns emerged: the thirteen TM7x-susceptible genomes, including XH001, span the two closely-related species *A. odontolyticus* and *A. meyeri* (>83% AAI to XH001) and a few unnamed strains ranging from 74 to 85% AAI to XH001 (Fig. S8a). These relationships were confirmed by a phylogenomic tree generated with PhyloPhlAn based on 387 concatenated core genes (Fig. S8b). The phylogenomic tree revealed an *A. odontolyticus* clade including four *A. odontolyticus* strains and *A*. sp. ICM39, which is sister to a monophyletic clade of the three nonpermissive strains, and another clade containing two *A. meyeri* strains and *A*. sp F0310.

**Fig. 5:**
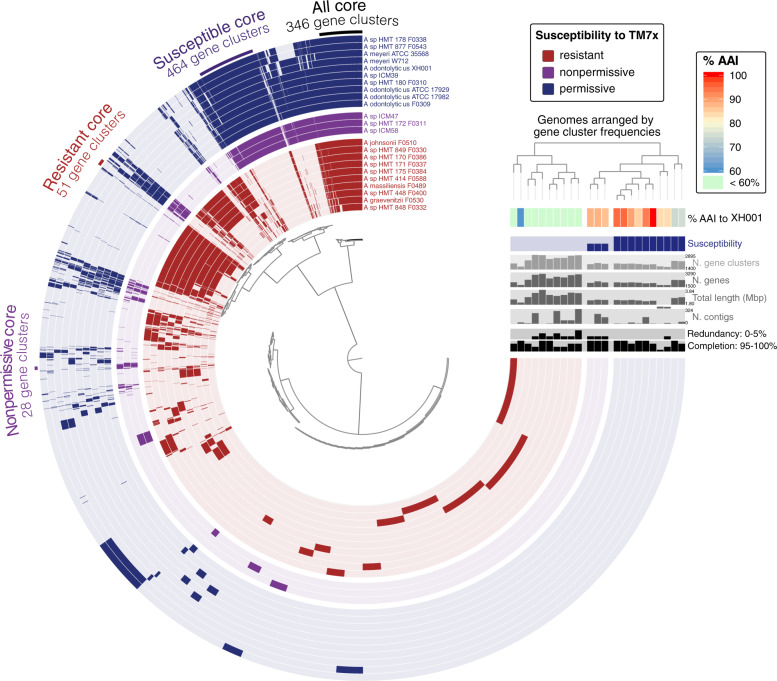
Pangenome of the experimentally tested *Actinomyces* strains. The central, radial dendrogram arranges each of 12,372 unique gene clusters (groups of putatively homologous genes) according to their presence/absence across genomes. Each concentric 270˚ layer represents a different genome, colored according to TM7x susceptibility, and is filled or left unfilled to mark which gene clusters are found in each genome. Layers are arranged by frequency of gene clusters, displayed as a dendrogram on the top righthand side of plot. Extending off the end of the plot show bar charts reporting various statistics for each genome and a heatmap showing average amino acid identity. The heatmap is also shown in Fig. S7 in higher magnification. Sets of key gene clusters are highlighted with a labeled arc spanning gene clusters of interest.

We then performed a pangenome analysis to compare the genome content of these strains (Fig. 5) to identify genomic signatures associated with different susceptibility to TM7x infection. By grouping genomes based on gene content (Fig. 5, top right dendrogram), the resistant strains (concentric layers colored red) are clearly separated from the susceptible strains (permissive (blue) and nonpermissive (purple); Fig. 5), agreeing closely with the phylogenomic tree (Fig. S8b). Remarkably, within the susceptible strains the nonpermissive strains (purple) form an internal subgroup distinct from permissive strains (blue) (Fig. 5). All phylogenomic analyses and AAI are consistent with the observed separation of groups (heatmap in Fig. 5), while the 16S rRNA gene phylogeny fails to indicate that the purple group of nonpermissive hosts is distinct (Fig. 1). As the susceptible strains span at least two phylogenetically classified species (*A. odontolyticus* and *A. meyeri*) and potentially other closely related but unnamed species, the genome grouping by gene content broadly reflects the previously observed phylogenomic and AAI distinctions (Figs. 5, S8). F0310 was the only strain that shifted places from being similar to *A. meyeri* species based on genome sequence (in phylogenomic tree) to being in middle of the *A. odontolyticus* species. Based on the gene content and phylogenomic tree, the nonpermissive genomes are a genetically distinct group most closely related to *A. odontolyticus* and less so to *A. meyeri*.

Furthermore, core gene clusters for the various groups can be readily discerned, with 346 gene clusters forming the core of all 23 genomes, 464 exclusively shared by all susceptible strains, and 51 and 28 gene clusters exclusively shared by the resistant and nonpermissive strains, respectively (Fig. 5, Table S2). For context, each genome contains ~1700–2800 gene clusters (Fig. 5, light gray bar chart on right). While most genomes are estimated to be nearly complete and a handful are closed, most of the genomes are not closed and may be missing genes for methodological rather than biological reasons (Fig. 5, bar charts of genome statistics). Yet, the correlation of gene content with response to TM7x raises the possibility that certain shared genomic features may underly the observed phenotypes.

### Comparative genomics reveal functional characteristics of different groups

We observed clades of strains defined by phylogeny and response to TM7x, e.g., permissive hosts. Ranking the predicted functions found across genomes for each TM7x response category (permissive, nonpermissive, or resistant) and combinations thereof can reveal functions enriched for each response type. The differentially enriched functions for these groups span multiple functional categories, from central metabolism to cell wall synthesis to regulation and recombination (Table 1).

**Table 1:** Enriched Pfam functions in resistant, susceptible, permissive, nonpermissive, and nonpermissive/resistant genomes. Only the top five gene functions are shown.

	Predicted Pfam function	Enrich. Score^b^	Adj. *q* value^c^	Observation in-group^a^		
				*R*	*NP*	*P*
Resistant	Mur ligase family, glutamate ligase domain	23	6.28E-04	10/10	0/3	0/10
	NADH:flavin oxidoreductase/NADH oxidase family	23	6.28E-04	10/10	0/3	0/10
	Thiamine pyrophosphokinase C terminal	23	6.28E-04	10/10	0/3	0/10
	Amidinotransferase ArcA	23	6.28E-04	10/10	0/3	0/10
	Bacitracin resistance protein BacA	23	6.28E-04	10/10	0/3	0/10
	Glycosyl transferase WecB/TagA/CpsF family	23	6.28E-04	0/10	3/3	10/10
Susceptible (permissive + nonpermissive)	C-terminal four TMM region of protein-O-mannosyltransferase	23	6.28E-04	0/10	3/3	10/10
	Dehydrogenase E1 component	23	6.28E-04	0/10	3/3	10/10
	Cytidylate kinase	23	6.28E-04	0/10	3/3	10/10
	Metallopeptidase family M81	23	6.28E-04	0/10	3/3	10/10
Permissive	Acyl-CoA dehydrogenase, C-terminal domain	13.78	0.013	2/10	1/3	10/10
	GlcNAc-PI de-N-acetylase	13.48	0.014	0/10	1/3	8/10
	Family 4 glycosyl hydrolase C-terminal domain	13.48	0.014	0/10	1/3	8/10
	Butirosin biosynthesis protein H, N-terminal	13.08	0.014	0/10	0/3	7/10
	Glycine zipper	13.08	0.014	0/10	0/3	7/10
Nonpermissive	Phage terminase, small subunit	16.74	0.005	1/10	3/3	0/10
	S-adenosylmethionine synthetase, N-terminal domain	14.6	0.009	0/10	2/3	0/10
	Domain of unknown function (DUF4391)	12.42	0.018	1/10	3/3	1/10
	Pectate lyase superfamily protein	10.03	0.047	1/10	3/3	2/10
	HsdM N-terminal domain	9.19	0.066	0/10	2/3	1/10
Nonpermissive + resistant	RmuC family	15.68	0.005	9/10	3/3	1/10
	Cytidine triphosphate (CTP) synthase	11.98	0.022	5/10	3/3	0/10
	TPM domain	10.55	0.037	10/10	3/3	4/10
	MafB19-like deaminase	10.55	0.037	10/10	3/3	4/10
	Tetracyclin repressor-like, C-terminal domain	10.55	0.037	10/10	3/3	4/10

^a^“Observation in-group” reports the fraction of genomes in each group that contained each gene cluster function. R = resistant, P = permissive, NP = nonpermissive.

^b^“Enrichment score” summarizes the number of in-group genomes containing this function vs. out-of-group genomes.

^c^“Adj. *q* value” is an FDR-corrected estimate of confidence.

For resistant vs. susceptible *Actinomyces*, numerous gene functions were exclusive to each (Table S2), potentially due to the strong genetic distinction between the two groups. Most pronounced of all functions were cell wall modification associated genes. Within the top five scored genes, we found Mur ligase family [34] and bacitracin resistance [35] proteins associated with resistant strains, and glycosyl transferase family [36] and O-mannosyltrasferase [37] proteins from susceptible strains (Table 1). These genes may directly or indirectly influence the TM7x attachment to the host. In addition, a key gene in the arginine deaminase (ADI) pathway, amidinotransferase *arcA*, was found in all ten of the resistant strains but none of the susceptible strains (Table 1). The ADI pathway can facilitate growth in acidic environments by increasing the pH, raising the possibility that TM7x, which encode a complete ADI pathway, could complement their ADI-less hosts [38]. Given the drastic oral pH shifts [39, 40] as well as localized pH stress from streptococcal neighbors [41], pH modulation and tolerance could be key for oral *Actinomyces* [40].

Permissive and nonpermissive genomes also contained distinctive functions (Table 1). For example, permissive strains are enriched for a GlcNAc-PI de-N-acetylase [42] and family 4 glycosyl hydrolase [43], which could be putatively involved in the hydrolysis of cell envelope glycoproteins, and may have the potential to regulate TM7x attachment levels. Interestingly, resistant and nonpermissive strains also share some functions not found in any permissive strains, such as a cytidine triphosphate (CTP) synthase.

### Amino acid variants reveal genes phylogenetically correlated with TM7x response

While comparing gene presence can reveal major traits that may be involved in the observed phenotypes, it cannot distinguish between subtle but potentially critical variations in the sequence of shared proteins. If TM7x susceptibility is not due to clade-specific genes but instead distinct sequence variants of certain core genes, those sequence variants should correlate with TM7x sensitivity.

**Fig. 6:**
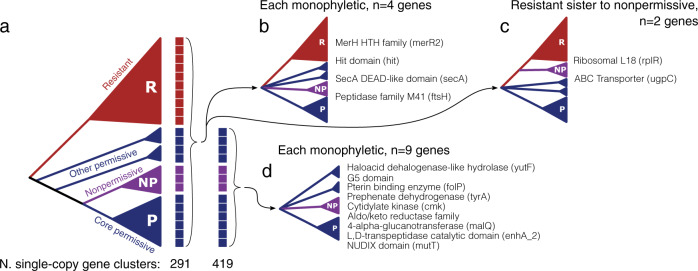
Gene trees from core gene clusters reveal gene variants that correlate with TM7x susceptibility. **a** Cartoon showing a simplified topology of the genome similarity dendrogram from Fig. 5, with the blue, purple, and red clades representing the permissive, nonpermissive, and resistant genomes respectively. Single-copy core gene clusters, those with only one gene sequence from each genome, core to all 23 genomes (first column of boxes, 291 gene clusters) and core to susceptible genomes (second column of boxes, 419 genes) were identified. For each gene cluster a phylogenetic tree was created and compared against three topologies of interest; gene clusters core to all genomes (**b** and **c**), and gene clusters core to susceptible genomes (**d**). Gene clusters core to all genomes could reveal each observed clade to be monophyletic with variable relationships (**b**) or place resistant sequences sister to those from nonpermissive hosts (**c**). The number over each arrow reports the number of gene clusters producing the illustrated topology. Polytomies represent either real polytomies or an unspecified hierarchy that preserves the monophyly of the illustrated clades. The text lists the predicted Pfam functions for each gene cluster.

Thus, we employed a phylogenetic approach to look for core genes with sequence variants that match the observed phenotypes. This is a powerful way to identify shared genes in a pangenome that are correlated with an ecological phenotype [44], though sometimes prone to false positives and noise. From each of the 291 and 419 gene clusters with a single copy in each of the 23 genomes and the 13 susceptible genomes, respectively (Fig. 6a), we created a phylogenetic tree and compared it against topologies that differentiated sequence variants from nonpermissive (purple) vs. permissive (blue) vs. resistant (red). Fifteen gene clusters produced such topologies that distinguished each response type (Fig. 6b–d). While some are almost certainly noise (e.g., ribosomal protein *rplR*), many functionally interesting genes are identified including several cell envelope-associated proteins like the protein translocase *secA*, the ABC transporter sn-glycerol-3-phosphate *ugpC*, and an L,D-transpeptidase (Fig. 6b–d). The genes listed here represent a relatively short list of hypotheses that await future experimental investigation before any confident assertions can be made about their relevance to *Actinomyces*/TM7x associations.

## Discussion

Host range is a key trait for many symbionts that can provide insights into what features are most critical for the association as well as reveal potentially different co-evolutionary strategies across a host range. The host selection process has not been well characterized for obligate bacterial parasites that target bacteria, likely reflecting the paucity of such unique organisms currently known. Thus, better understanding the TM7x/host relationship offers valuable detail and breadth to the study of bacterial/bacterial symbioses.

The lifecycle of most obligate parasites can be organized into a two-step pattern: Host/parasite selection/binding followed by infection (e.g., replication and maturation) [9]. For example, parasites use receptor-binding proteins or various secretion systems to initially adhere to their hosts [45–48], followed by post-binding steps such as replication which require the parasites to overcome host environment and defense mechanisms [47, 49–51]. Host range can be defined by one or both of these stages, where parasite success can depend on the existence of proper surface-binding proteins to dock on one specific (narrow) or multiple (broad) hosts [11]. Our study showed TM7x has intermediate host range, as it is restricted to the genus *Actinomyces* but can infect multiple species within *Actinomyces*. This range is still more restricted than other predatory bacteria such as *Bdellovibrio*, which prey on taxonomically diverse gram-negative bacteria [5, 10], and potentially more confined than *Micavibrio*, which have variably-reported host ranges [52, 53].

However, within this intermediate host range we found that the hosts are further distinguished by their initial response to TM7x, whether they crash or grow normally. We hypothesize that resistant strains may lack docking sites (e.g., receptor proteins or cell wall components) for TM7x as FISH performed on these resistant strains during growth did not reveal any TM7x attachment (Fig. S7). However, we did not explicitly test initial host binding. Within the susceptible hosts, all are likely to have suitable docking sites, as TM7x attachment was seen microscopically (Figs. 4, S2, S7). However, permissive and nonpermissive strains could potentially be distinguished by the amount or nature of surface-displayed docking molecules, as reflected by our phase-contrast imaging where the majority of the permissive strains had many TM7x on a single bacterial host (Fig. S2). The nonpermissive strains could also have post-attachment mechanisms that ameliorate TM7x-induced stress and death. In this scenario, the molecular mechanisms of how host range is determined (susceptible vs. resistant) and the nature of the relationship (permissive vs. nonpermissive) are separate events of the TM7x lifecycle, similar to the current hypothesis of epibiotic predation [54].

Comparative genomics revealed both cell surface and intracellular proteins that are differentially enriched in susceptible vs. resistant and permissive vs. nonpermissive strains (Table 1). Our previous transcriptomic analysis and recent study based on predicted metabolic complementarity between TM7x and XH001 similarly identified the importance of cell surface and cell wall components in the TM7x/XH001 relationship [38, 55]. Consistent with this idea, our analysis showed that most of the groups were enriched with different cell wall modifying genes at the carbohydrate and peptidoglycan modules (Table 1). Also, we identified genes in resistant and nonpermissive strains that encode intracellularly localized proteins like CTP synthase which could minimize the stress of hosting TM7x. However, further experimental work is required to verify their roles in *Actinomyces*-TM7x growth dynamics.

Multiple strains of Saccharibacteria were cultivated recently on *Actinomyces* as well as on other actinobacteria such as *Pseudopropionibacterium propionicum* and *Cellulosimicrobium cellulans* [32, 56]. These reports show that different Saccharibacteria lineages can associate with different host taxa. Saccharibacteria strains (e.g., HMT-346) that are taxonomically distant from TM7x (HMT-952) can grow on *Actinomyces* spp. that are incapable of supporting the growth of TM7x. In comparison, strains (e.g., HMT-352) that are more closely related to TM7x can grow on the same host as TM7x, such as *A. odontolyticus* [32]. However, these studies tested only a limited set of potential hosts for these Saccharibacteria and more thorough work is needed to better compare their host ranges.

Outside of bacteria, insights from archaeal/archaeal symbioses suggest that Saccharibacteria may contain additional host relationship strategies beyond those employed by TM7x. While all have reduced genomes and rely on a host for critical resources, each characterized archaeal symbiont has a unique relationship with its host that appears to be based upon presumably independent co-evolution [57–61]. If the same is true for Saccharibacteria, then many more exciting saccharibacterial relationships remain to be discovered. In terms of host range, however, archaeal/archaeal symbionts may differ from TM7x, as Nanoarchaeota and Nanohaloarchaeota collectively associate with diverse hosts, yet clonal lineages appear to pair with single host species [57, 58] although closely-related lineages can pair with different host strains of the same species [62].

This study looked at the interactions of one TM7x strain with multiple viable hosts, but future studies are needed to address host/symbiont evolution more comprehensively by including additional strains of Saccharibacteria, as their host ranges cannot be surmised from this study alone. Because Saccharibacteria have many more metabolic and molecular capabilities than phages, the network of interactions determining the nature of the symbiotic association could be substantially more complicated. Further, the relationships between Saccharibacteria and Actinobacteria in their native oral habitats require direct investigation. While microscopy confirms the epibiotic association is conserved [32, 55, 56], the potentially diverse in situ interactions could significantly alter the host/symbiont relationships. As the majority of the diverse and ubiquitous CPR phyla are thought to engage in similarly obligate symbioses [63, 64], the extent of host range for CPR organisms could have tremendous impact on our understanding of the role such bacteria/bacteria associations play in their respective environments, from the oral microbiome to aquatic and terrestrial habitats. Beyond TM7x and the CPR, our work underscores the importance of investigating symbiotic associations across a range of hosts, by demonstrating how a single obligate symbiont’s impact varied from deleterious to neutral across several different hosts.

## Supplementary information

Supplementary Information

Figure S1

Figure S2

Figure S3

Figure S4

Figure S5

Figure S6

Figure S7

Figure S8

Table S1

Table S2

## References

[1] Douglas AE. The symbiotic habit. USA: Princeton Univ. Press; 2010.

[2] Archibald J. One plus one: symbiosis and the evolution of complex life. Great Britian: Oxford Univ. Press; 2014.

[3] Seckbach J. Symbiosis: mechanisms and model systems. New York, Boston, Dordrecht, London, Moscow: Kluwer Academic Publisher; 2002.

[4] von Dohlen CD, Kohler S, Alsop ST, McManus WR (2001). Mealybug β-proteobacterial endosymbionts contain γ-proteobacterial symbionts. Nature.

[5] Sockett RE (2009). Predatory lifestyle of Bdellovibrio bacteriovorus. Annu Rev Microbiol.

[6] Guerrero R, Pedros-Alio C, Esteve I, Mas J, Chase D, Margulis L (1986). Predatory prokaryotes: predation and primary consumption evolved in bacteria. Proc Natl Acad Sci USA.

[7] He X, McLean JS, Edlund A, Yooseph S, Hall AP, Liu S-Y (2015). Cultivation of a human-associated TM7 phylotype reveals a reduced genome and epibiotic parasitic lifestyle. Proc Natl Acad Sci USA.

[8] Bor B, McLean JS, Foster KR, Cen L, To TT, Serrato-Guillen A (2018). Rapid evolution of decreased host susceptibility drives a stable relationship between ultrasmall parasite TM7x and its bacterial host. Proc Natl Acad Sci.

[9] Sieber M, Gudelj I (2014). Do-or-die life cycles and diverse post-infection resistance mechanisms limit the evolution of parasite host ranges. Ecol Lett..

[10] Dashiff A, Junka RA, Libera M, Kadouri DE (2011). Predation of human pathogens by the predatory bacteria Micavibrio aeruginosavorus and Bdellovibrio bacteriovorus. J Appl Microbiol.

[11] de Jonge PA, Nobrega FL, Brouns SJJ, Dutilh BE (2019). Molecular and evolutionary determinants of bacteriophage host range. Trends Microbiol..

[12] Bor B, Bedree JK, Shi W, McLean JS, He X (2019). Saccharibacteria (TM7) in the Human Oral Microbiome. J Dent Res.

[13] Baker JL, Bor B, Agnello M, Shi W, He X (2017). Ecology of the oral microbiome: beyond bacteria. Trends Microbiol.

[14] Brown CT, Hug LA, Thomas BC, Sharon I, Castelle CJ, Singh A (2015). Unusual biology across a group comprising more than 15% of domain Bacteria. Nature.

[15] Hug LA, Baker BJ, Anantharaman K, Brown CT, Probst AJ, Castelle CJ (2016). A new view of the tree of life. Nat Microbiol..

[16] McLean JS, Liu Q, Bor B, Bedree JK, Cen L, Watling M (2016). Draft Genome Sequence of *Actinomyces odontolyticus* subsp. *actinosynbacter* Strain XH001, the Basibiont of an Oral TM7 Epibiont. Genome Announc.

[17] Bor B, Poweleit N, Bois JS, Cen L, Bedree JK, Zhou ZH (2016). Phenotypic and physiological characterization of the epibiotic interaction between TM7x and its basibiont actinomyces. Micro Ecol..

[18] Stamatakis A, Hoover P, Rougemont J. A rapid bootstrap algorithm for the RAxML web servers. Syst Biol. 2008;57(Oct):758–71. 10.1080/10635150802429642 18853362

[19] Hyatt D, Chen G-L, LoCascio PF, Land ML, Larimer FW, Hauser LJ (2010). Prodigal: prokaryotic gene recognition and translation initiation site identification. BMC Bioinforma..

[20] Buchfink B, Xie C, Huson DH (2015). Fast and sensitive protein alignment using DIAMOND. Nat Methods.

[21] Segata N, Börnigen D, Morgan XC, Huttenhower C (2013). PhyloPhlAn is a new method for improved phylogenetic and taxonomic placement of microbes. Nat Commun..

[22] Paradis E, Schliep K. ape 5.0: an environment for modern phylogenetics and evolutionary analyses in R. Bioinformatics. 2019;35(Feb):526–8. 10.1093/bioinformatics/bty633 30016406

[23] Revell LJ (2012). phytools: an R package for phylogenetic comparative biology (and other things). Methods Ecol Evolution.

[24] Delmont TO, Eren AM (2018). Linking pangenomes and metagenomes: the *Prochlorococcus* metapangenome. PeerJ.

[25] Lee MD. GToTree: a user-friendly workflow for phylogenomics. Bioinformatics. 2019;35(Oct):4162–4. 10.1093/bioinformatics/btz188 PMC6792077 30865266

[26] van Dongen SM. Graph clustering by flow simulation [Internet]. Ph.D Thesis, University of Utrecht, Utrecht University Repository: http://dspace.library.uu.nl/handle/1874/848; 2000. Utrecht University Repository: http://dspace.library.uu.nl/handle/1874/848.

[27] Altschul SF, Gish W, Miller W, Myers EW, Lipman DJ (1990). Basic local alignment search tool. J Mol Biol.

[28] Shaiber A, Willis AD, Delmont TO, Roux S, Chen L-X, Schmid AC, et al. Functional and genetic markers of niche partitioning among enigmatic members of the human oral microbiome [Internet]. Microbiology. 2020 [cited 2020 May 16]. 10.1101/2020.04.29.069278. 10.1186/s13059-020-02195-w PMC7739484 33323122

[29] Price MN, Dehal PS, Arkin AP. FastTree 2 – approximately maximum-likelihood trees for large alignments. PLoS ONE. 2010;5(Mar):e9490. 10.1371/journal.pone.0009490 PMC2835736 20224823

[30] Papkou A, Gokhale CS, Traulsen A, Schulenburg H (2016). Host–parasite coevolution: why changing population size matters. Zoology.

[31] Wangersky PJ (1978). Lotka-volterra population model. Ann Rev Ecol Syst.

[32] Cross KL, Campbell JH, Balachandran M, Campbell AG, Cooper SJ, Griffen A (2019). Targeted isolation and cultivation of uncultivated bacteria by reverse genomics. Nat Biotechnol..

[33] Eswara PJ, Ramamurthi KS (2017). Bacterial cell division: nonmodels poised to take the spotlight. Annu Rev Microbiol.

[34] Smith CA (2006). Structure, function and dynamics in the mur family of bacterial cell wall ligases. J Mol Biol.

[35] Ghachi ME, Derbise A, Bouhss A, Mengin-Lecreulx D (2005). Identification of multiple genes encoding membrane proteins with Undecaprenyl Pyrophosphate Phosphatase (UppP) Activity in *Escherichia coli*. J Biol Chem.

[36] Kattke MD, Gosschalk JE, Martinez OE, Kumar G, Gale RT, Cascio D, et al. Structure and mechanism of TagA, a novel membrane-associated glycosyltransferase that produces wall teichoic acids in pathogenic bacteria. PLoS Pathog. 2019;15(Apr):e1007723. 10.1371/journal.ppat.1007723 PMC6493773 31002736

[37] Becker K, Haldimann K, Selchow P, Reinau LM, Dal Molin M, Sander P (2017). Lipoprotein Glycosylation by Protein-O-Mannosyltransferase (MAB_1122c) contributes to low cell envelope permeability and antibiotic resistance of mycobacterium abscessus. Front Microbiol..

[38] Bernstein DB, Dewhirst FE, Segrè D (2019). Metabolic network percolation quantifies biosynthetic capabilities across the human oral microbiome. Elife.

[39] Kleinberg I (1967). Effect of varying sediment and glucose concentrations on the pH and acid production in human salivary sediment mixtures. Arch Oral Biol.

[40] Edlund A, Yang Y, Yooseph S, Hall AP, Nguyen DD, Dorrestein PC (2015). Meta-omics uncover temporal regulation of pathways across oral microbiome genera during in vitro sugar metabolism. ISME J..

[41] Wilbert SA, Mark Welch JL, Borisy GG. Spatial ecology of the human tongue dorsum microbiome. SSRN J. [Internet]. 2019 [cited 2020 Feb 3]. https://www.ssrn.com/abstract=3438369. 10.1016/j.celrep.2020.02.097 PMC7179516 32209464

[42] Deli A, Koutsioulis D, Fadouloglou VE, Spiliotopoulou P, Balomenou S, Arnaouteli S (2010). LmbE proteins from Bacillus cereus are de-N-acetylases with broad substrate specificity and are highly similar to proteins in Bacillus anthracis: LmbE proteins from Bacillus cereus. FEBS J..

[43] Vermassen A, Leroy S, Talon R, Provot C, Popowska M, Desvaux M (2019). Cell wall hydrolases in bacteria: insight on the diversity of cell wall amidases, glycosidases and peptidases toward peptidoglycan. Front Microbiol..

[44] Shapiro BJ, Friedman J, Cordero OX, Preheim SP, Timberlake SC, Szabó G (2012). Population genomics of early events in the ecological differentiation of bacteria. Science.

[45] Evans KJ, Lambert C, Sockett RE (2007). Predation by Bdellovibrio bacteriovorus HD100 Requires Type IV Pili. J Bacteriol.

[46] Dowah ASA, Clokie MRJ (2018). Review of the nature, diversity and structure of bacteriophage receptor binding proteins that target Gram-positive bacteria. Biophys Rev..

[47] Moya A, Peretó J, Gil R, Latorre A (2008). Learning how to live together: genomic insights into prokaryote–animal symbioses. Nat Rev Genet.

[48] Dale C, Moran NA (2006). Molecular interactions between bacterial symbionts and their hosts. Cell.

[49] Rittenberg SC, Langley D (1975). Utilization of nucleoside monophosphates per Se for intraperiplasmic growth of Bdellovibrio bacteriovorus. J Bacteriol.

[50] Rostøl JT, Marraffini L (2019). (Ph)ighting phages: how bacteria resist their parasites. Cell Host Microbe.

[51] Ruby EG, McCabe JB, Barke JI (1985). Uptake of intact nucleoside monophosphates by Bdellovibrio bacteriovorus 109J. J Bacteriol.

[52] Kadouri D, Venzon NC, O’Toole GA (2007). Vulnerability of pathogenic biofilms to Micavibrio aeruginosavorus. Appl Environ Microbiol.

[53] Wang Z, Kadouri DE, Wu M (2011). Genomic insights into an obligate epibiotic bacterial predator: Micavibrio aeruginosavorus ARL-13. BMC Genom..

[54] Pérez J, Moraleda-Muñoz A, Marcos-Torres FJ, Muñoz-Dorado J (2016). Bacterial predation: 75 years and counting!: bacterial predation. Environ Microbiol..

[55] McLean JS, Bor B, Kerns KA, Liu Q, To TT, Solden L (2020). Acquisition and adaptation of ultra-small parasitic reduced genome bacteria to mammalian hosts. Cell Rep..

[56] Bor B, Collins AJ, Murugkar PP, Balasubramanian S, To TT, Hendrickson EL (2020). Insights obtained by culturing saccharibacteria with their bacterial hosts. J Dent Res..

[57] Huber H, Hohn MJ, Rachel R, Fuchs T, Wimmer VC, Stetter KO (2002). A new phylum of Archaea represented by a nanosized hyperthermophilic symbiont. Nature.

[58] Podar M, Makarova KS, Graham DE, Wolf YI, Koonin EV, Reysenbach A-L (2013). Insights into archaeal evolution and symbiosis from the genomes of a nanoarchaeon and its inferred crenarchaeal host from Obsidian Pool, Yellowstone National Park. Biol Direct.

[59] Wurch L, Giannone RJ, Belisle BS, Swift C, Utturkar S, Hettich RL (2016). Genomics-informed isolation and characterization of a symbiotic Nanoarchaeota system from a terrestrial geothermal environment. Nat Commun..

[60] Hamm JN, Erdmann S, Eloe-Fadrosh EA, Angeloni A, Zhong L, Brownlee C (2019). Unexpected host dependency of Antarctic Nanohaloarchaeota. Proc Natl Acad Sci USA.

[61] St. John E, Liu Y, Podar M, Stott MB, Meneghin J, Chen Z (2019). A new symbiotic nanoarchaeote (Candidatus Nanoclepta minutus) and its host (Zestosphaera tikiterensis gen. nov., sp. nov.) from a New Zealand hot spring. Syst Appl Microbiol.

[62] Munson-McGee JH, Field EK, Bateson M, Rooney C, Stepanauskas R, Young MJ. Nanoarchaeota, their sulfolobales host, and nanoarchaeota virus distribution across yellowstone national park hot springs. Appl Environ Microbiol. 2015;81(Nov):7860–8. 10.1128/AEM.01539-15 PMC4616950 26341207

[63] Gong J, Qing Y, Guo X, Warren A (2014). “Candidatus Sonnebornia yantaiensis”, a member of candidate division OD1, as intracellular bacteria of the ciliated protist Paramecium bursaria (Ciliophora, Oligohymenophorea). Syst Appl Microbiol.

[64] Castelle CJ, Banfield JF (2018). Major new microbial groups expand diversity and alter our understanding of the tree of life. Cell.

