# Supplementary material for: The saccharibacterium TM7x elicits differential responses across its host range
Source: ISME J. 2020 Aug 24;14(12):3054–67. doi: 10.1038/s41396-020-00736-6 (PMC7784981; doi:10.1038/s41396-020-00736-6)
Supplement: Supplementary file 1 — Supplementary Information [file 41396_2020_736_MOESM1_ESM.docx]

**Supplementary Information and Figure Legend**

**Material and Methods**

**TM7x isolation**

TM7x cells were isolated away from the bacterial host (XH001) using a previously developed method [1]. Briefly, overnight grown TM7x-XH001 coculture was centrifuged at 3,500 x g to pellet host bacteria and attached TM7x cells. Supernatant with free-floating TM7x was passed through a 0.45-micron Stericups (Millipore, Filtration System) to collect TM7x cells. Vacuum was applied at the lowest setting possible to avoid forcing large host bacteria through the filter membrane. The collected cells were ultracentrifuged at 80,000 x g for 80 minutes. The pellets were resuspended in 1 mL of fresh BHI to create a concentrated suspension of TM7x cells independent of their hosts. These isolated TM7x cells have not been able to grow on their own even in rich media as we tested them for a minimum of 48 hours at 37°C. This also functioned as a test to check for XH001 contamination. We further assessed purity of the TM7x microscopically and by examining the morphology of the host bacteria after infection. If original XH001 host passed through the membrane, then all the different bacterial hosts that we tested will have same morphology after crash/recovery.

**Fluorescence In Situ Hybridization (FISH) imaging**

FISH was carried out as previously described [2]. Mono or coculture cells were fixed by 4 % formaldehyde for 3 h and treated with 2 mg/ml lysozyme. Each coculture cells were harvested from the re-infection experiment after establishing the infection and cocultivation. Fixed cells were resuspended in hybridization buffer and incubated at 37 °C for 30 min before staining with TM7x-specific probe. Cells were visualized by a Leica SPE I inverted confocal microscope equipped with an ACS APO 100x/1.15 oil CS immersion objective. For the coculture cells, only those late stage (after multiple passages), already established cocultures were imaged.

**Host cell length analysis**

Cell length of all susceptible *Actinomyces* were analyzed according to previous method [2]. Briefly, phase contrast images were acquired during infection for each host with and without TM7x infection (three independent experiments). Cells were imaged using a Nikon Eclipse E400 microscope equipped with a Nikon Plan Fluor Å~100/1.30 oil immersion objective. Images were processed using tools available in SciPy version 0.10.0 [3] and scikit-image version 0.14.0 [4] to obtain the length. When the cells become too long, our method struggles to distinguish one cell from the next since number of cells touching each other increases. This is an inherent drawback that many image analysis tools face, and we were careful to prepare our samples so that cells are suitably dispersed. The individuals were blinded when analyzing the images. Only after completing the images that we assigned groups. Cell length density plots and subsequent statistical analyses (t-test and Kolmogorov-Smirnov test) were generated in R (<https://www.r-project.org/>). P values are reported in Figure S3.

**Figure Legends**

**Figure S1.** Re-infection of bacterial hosts. (a) *Actinomyces* infection diagram, and naming based on observed phenotypes. (b) Isolated TM7x cells were added to XH001 cells at passage 0, and cell density (blue, circles) and TM7x scores (red, squares) were monitored during subsequent passages. TM7x scores are qualitative measurement of both attached and free-floating TM7x cells. XH001 alone control is shown in gray triangles. The same experiment as (b) was carried out with all hosts identified and graphed individually in Figure 2. From these experiments, cell density measurements (c) and initial increase of TM7x scores (d) were plotted. (c and d) All the permissive hosts are shown in gray color while three nonpermissive hosts are shown in blue (F0311), green (ICM47) and red (ICM58). (e) Bacterial host cell length was measured for monoculture (gray) and cocultures (red) after infection by TM7x. Gray and red lines indicate the average cell length.

**Figure S2.** Phase contrast images of the twelve susceptible bacteria. (a-l) Images on the top rows are hosts grown without TM7x and bottom rows are hosts infected with TM7x and imaged during the cell growth-crash passages. All scale bars are 10 μm. Permissive (a-i) and nonpermissive (j-l) hosts contain TM7x cells; some of the attached, free-floating and long TM7x cells are indicated by red arrows. Many times, when hosts are going under growth-crash, we observed many TM7x infecting a single host that looks very ‘fuzzy’.

**Figure S3.** Cell length density distribution of the twelve susceptible bacteria. (a-l) Using R environment, twelve hosts with (red) and without (black) TM7x were plotted. X-axis is cell length in μm and Y-axis is density of the cell length across each population. Median is indicated by the dotted red or black lines. Student t-test and Kolmogorov–Smirnov (ks) test were conducted and the resulting p-values are displayed in the upper right corner boxes. Number of cells quantified is shown by n, and median is graphed in Figure S1d. Permissive (a-i) and nonpermissive (j-l) hosts are shown accordingly.

**Figure S4.** Growth-crash experiment that is the same as in Figure S1b, but this time increasing doses of TM7x were added to fixed number of XH001. First column is cell density measurement (blue, circles) and TM7x scores (red, squires) plotted across multiple passages. Second column is the total colony forming units (blue, circles) and irregular colony forming units (red, squares) of the same experiments in first column if plotted. Total colony numbers reflect overall population of the XH001 while irregular colony number reflect infected XH001 cells.

**Figure S5.** The same TM7x dose dependent experiment as in Figure S3 was carried out but the bacterial host used was W712.

**Figure S6.** The same TM7x dose dependent experiment as in Figure S3 and S4 was carried out but the bacterial host is ICM47. Infected ICM47 cells did not show obvious irregular colonies and therefore irregular colony numbers were not plotted. Total colony forming units are plotted in Figure 3.

**Figure S7.** The same FISH experiment was carried out in all susceptible and resistant strains. Growth-crasher (a-g) and resistant (h-i) bacterial hosts were stained with TM7x (green) specific DNA probe and nucleic acid stain syto9 (red). For resistant strain, only representative images are shown. Scale bars are 5 μm.

**Figure S8.** (a) Heatmap of amino acid identity between the 23 *Actinomyces* genomes. Exact AAI values are printed in each cell. Genomes are ordered as in Figure 5, and permissive (blue), nonpermissive (purple) and resistant (red) strains are indicated. (b) Cophylogeny illustrating the close relationship between a phylogenomic tree based on concatenated core genes (left tree) and the pangenome’s dendrogram arranging genomes by gene cluster frequency (right). All branches in the phylogenomic tree received 100% bootstrap support. Lines linking the tips are colored based on response type to TM7x, as in Figure 5. The one strain differently placed between the trees is bolded.

**Table S1**. Comprehensive list of Actinomyces and other bacterial strains tested for TM7x re-infection assay with growth conditions and strain sources.

**Table S2**. Summary information of the pangenome and functional enrichment analyses. The first tab contains data for all gene clusters in the *Actinomyces* pangenome, including their membership in the various sets (e.g. “Susceptible core”), Pfam, GO, and KEGG annotations. The second tab contains the functional enrichment scores for all predicted Pfam functions, including the genome group(s) in which the function was predicted to be enriched as well as the statistical summary of each function’s occurrence in those genomes

**References**

1. Bor B, McLean JS, Foster KR, Cen L, To TT, Serrato-Guillen A, et al. Rapid evolution of decreased host susceptibility drives a stable relationship between ultrasmall parasite TM7x and its bacterial host. *Proceedings of the National Academy of Sciences* 2018; **115**: 12277–12282.

2. Bor B, Poweleit N, Bois JS, Cen L, Bedree JK, Zhou ZH, et al. Phenotypic and Physiological Characterization of the Epibiotic Interaction Between TM7x and Its Basibiont Actinomyces. *Microb Ecol* 2016; **71**: 243–255.

3. E Jones, E Oliphant, P Peterson, et al. SciPy: Open Source Scientific Tools for Python. http://www.scipy.org/. .

4. S Walt, JL Schonberger, J Nunez-Iglesias, et al, and the Scikit-image contributors. Scikit-image: Image processing in Pythong. PeerJ 2:e453. http://dx.doi.org/10.7717/peerj.453.
