# Supplementary figures and images for: The saccharibacterium TM7x elicits differential responses across its host range
Source: ISME J. 2020 Aug 24;14(12):3054–67. doi: 10.1038/s41396-020-00736-6 (PMC7784981; doi:10.1038/s41396-020-00736-6)

Figure S1.

a

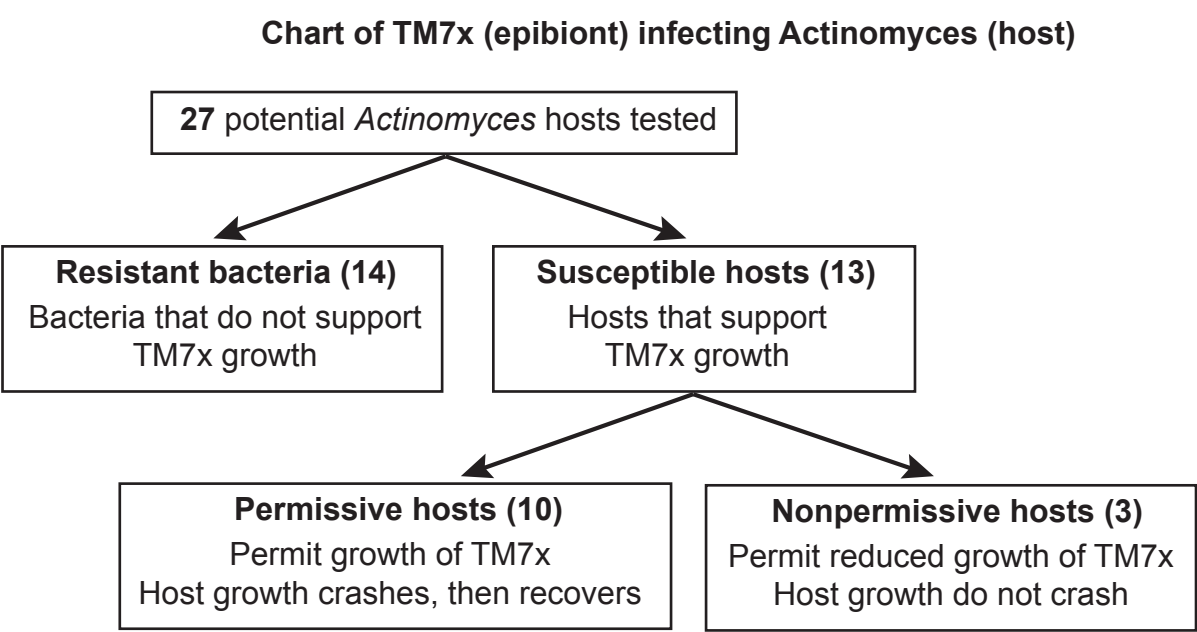

b

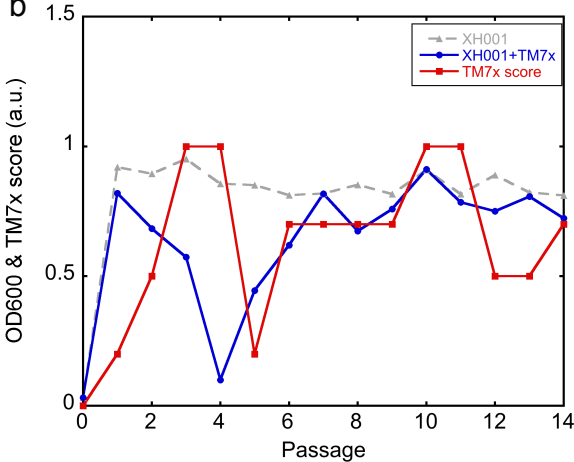

c

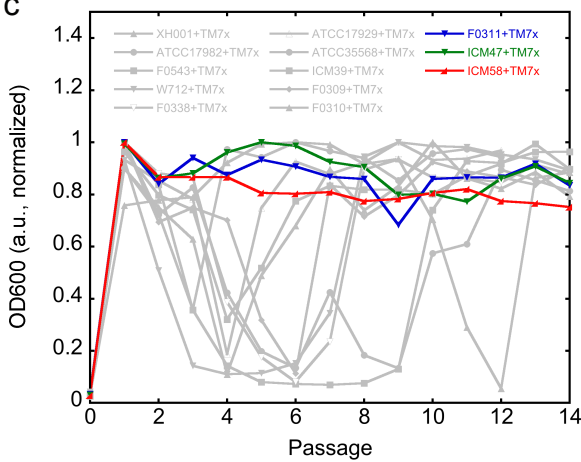

d

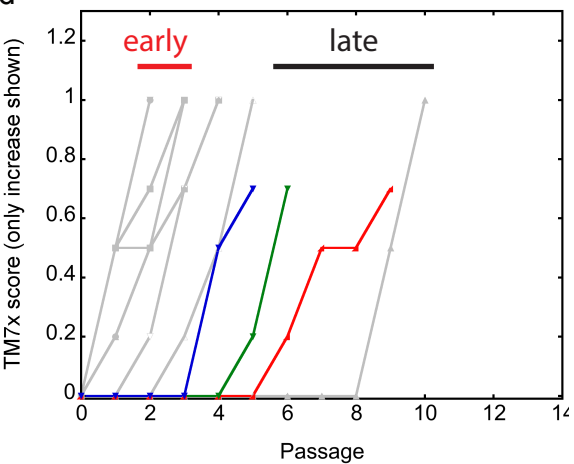

e

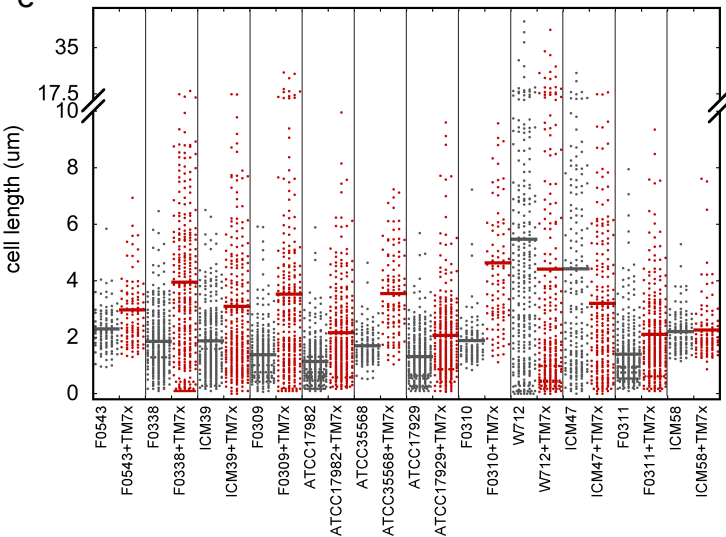

Supplement: Supplementary file 2 — Figure S1 [file 41396_2020_736_MOESM2_ESM.pdf]

Figure S2.

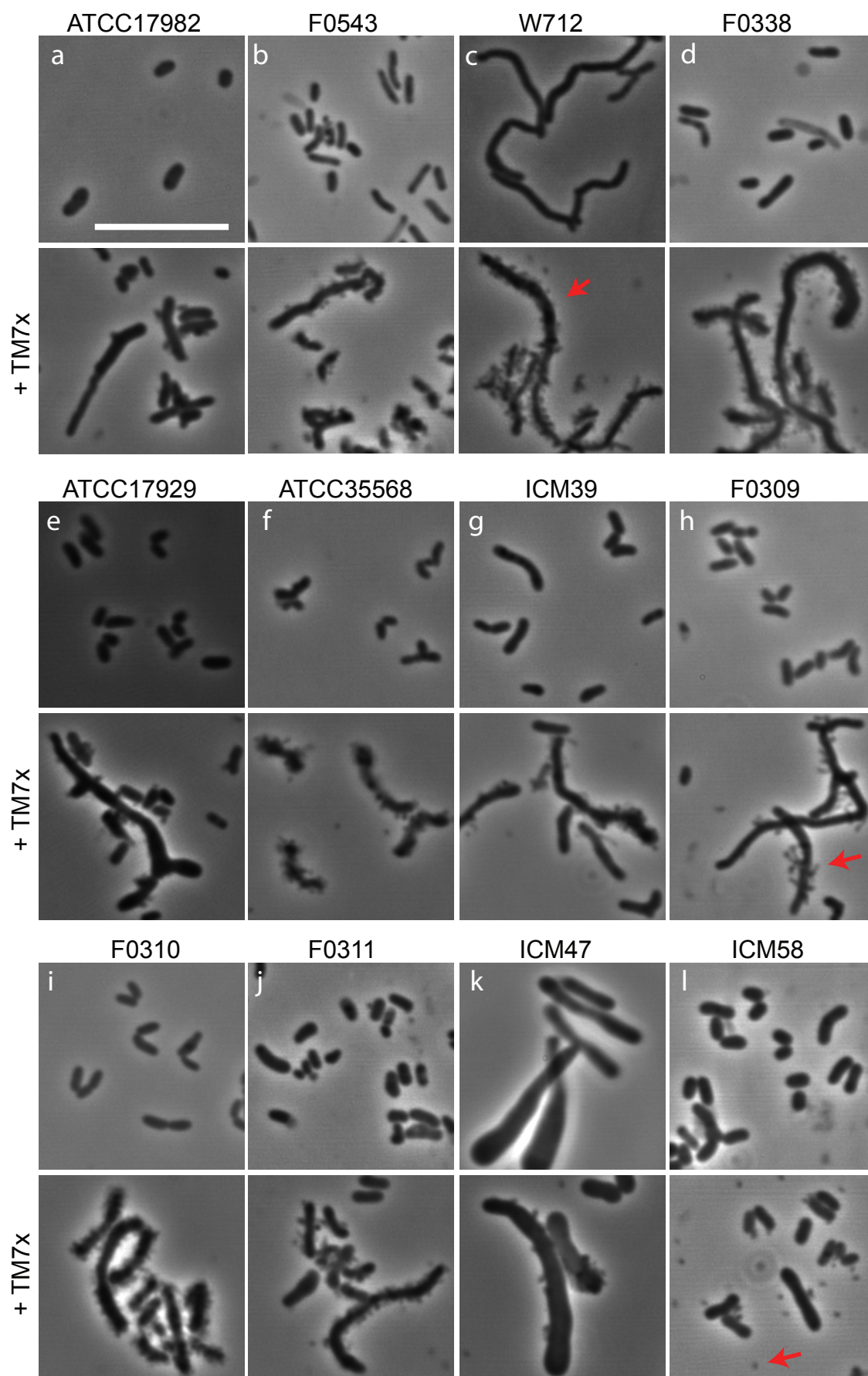

Supplement: Supplementary file 3 — Figure S2 [file 41396_2020_736_MOESM3_ESM.pdf]

Figure S3.

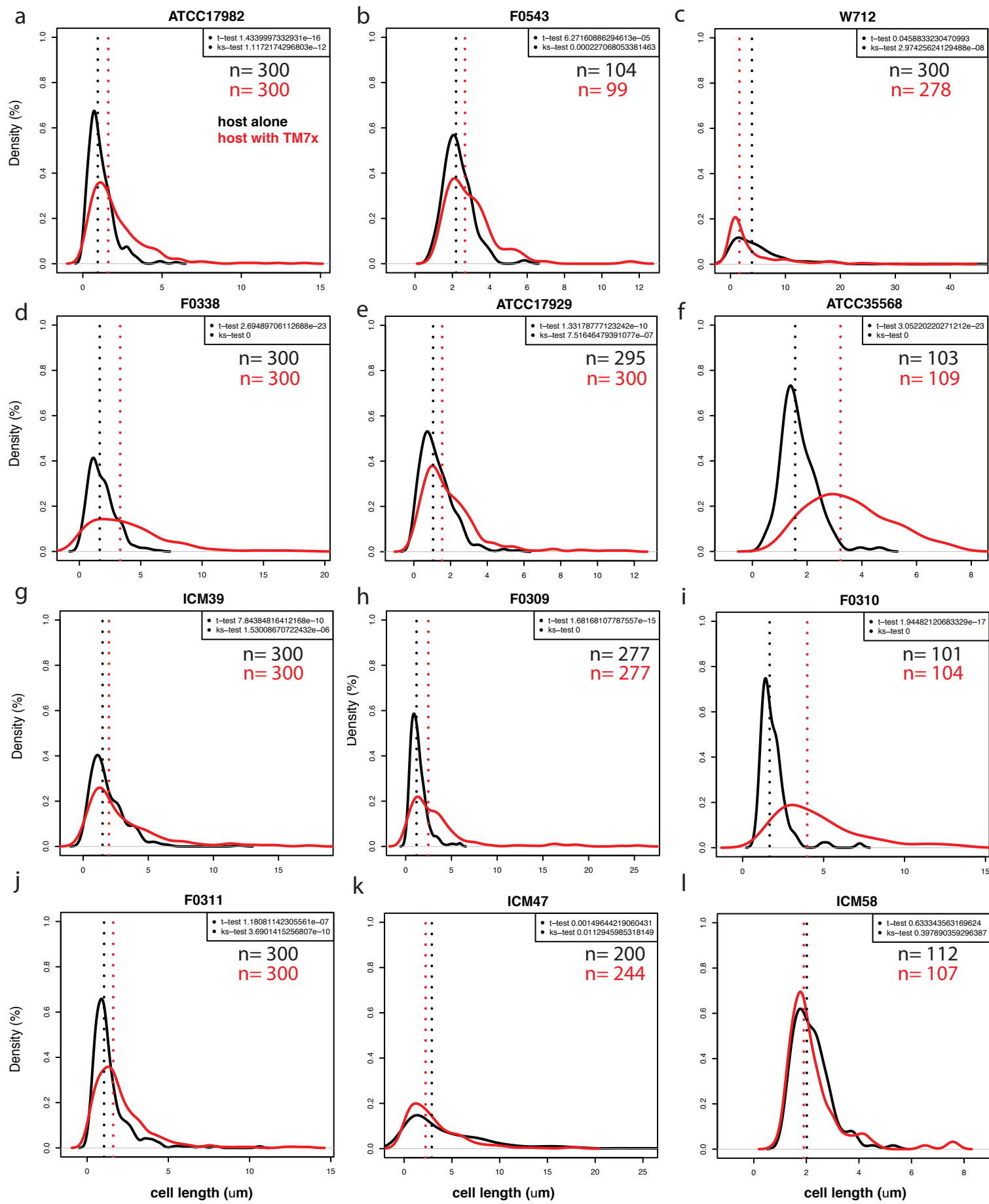

Supplement: Supplementary file 4 — Figure S3 [file 41396_2020_736_MOESM4_ESM.pdf]

Figure S4.

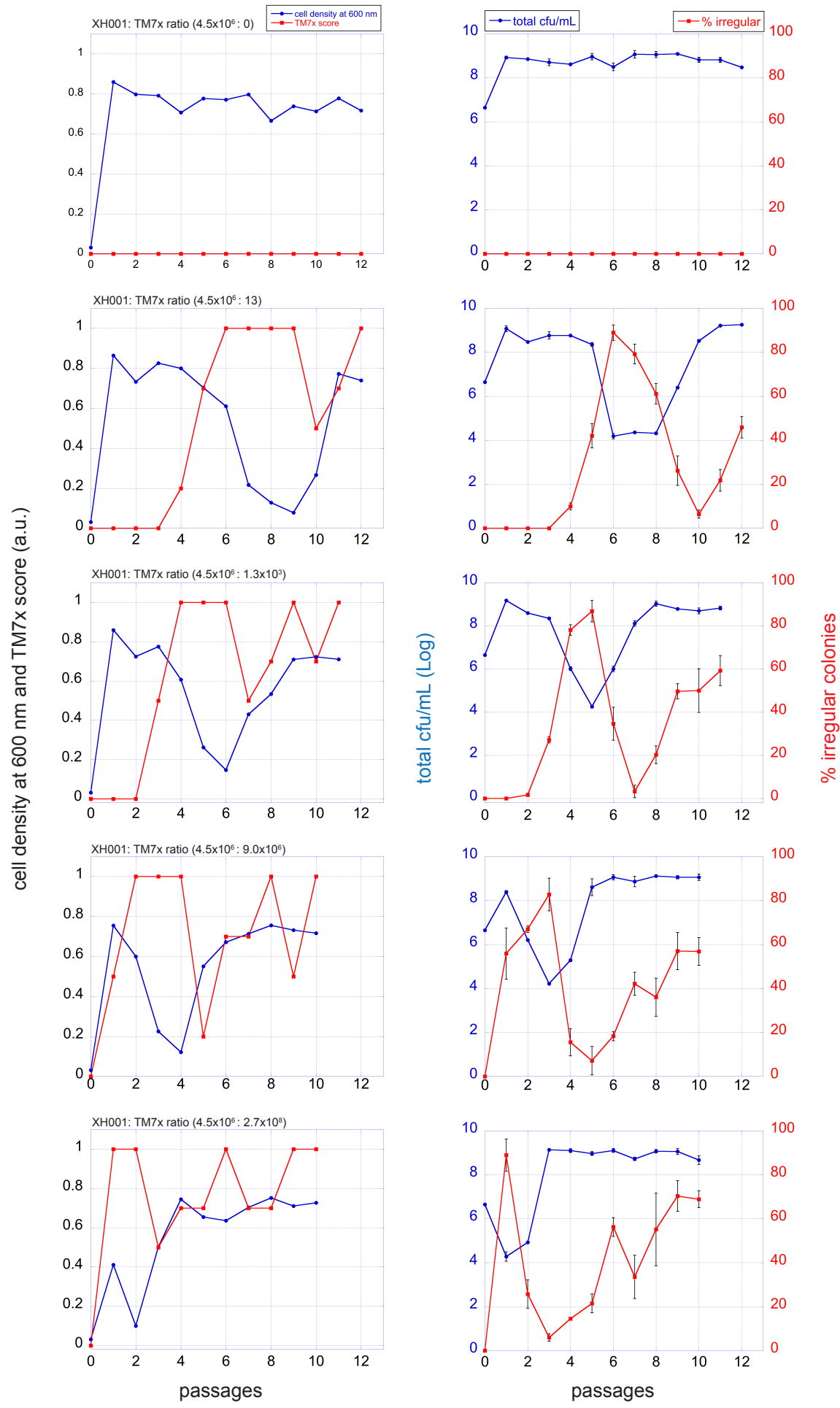

Supplement: Supplementary file 5 — Figure S4 [file 41396_2020_736_MOESM5_ESM.pdf]

Figure S5.

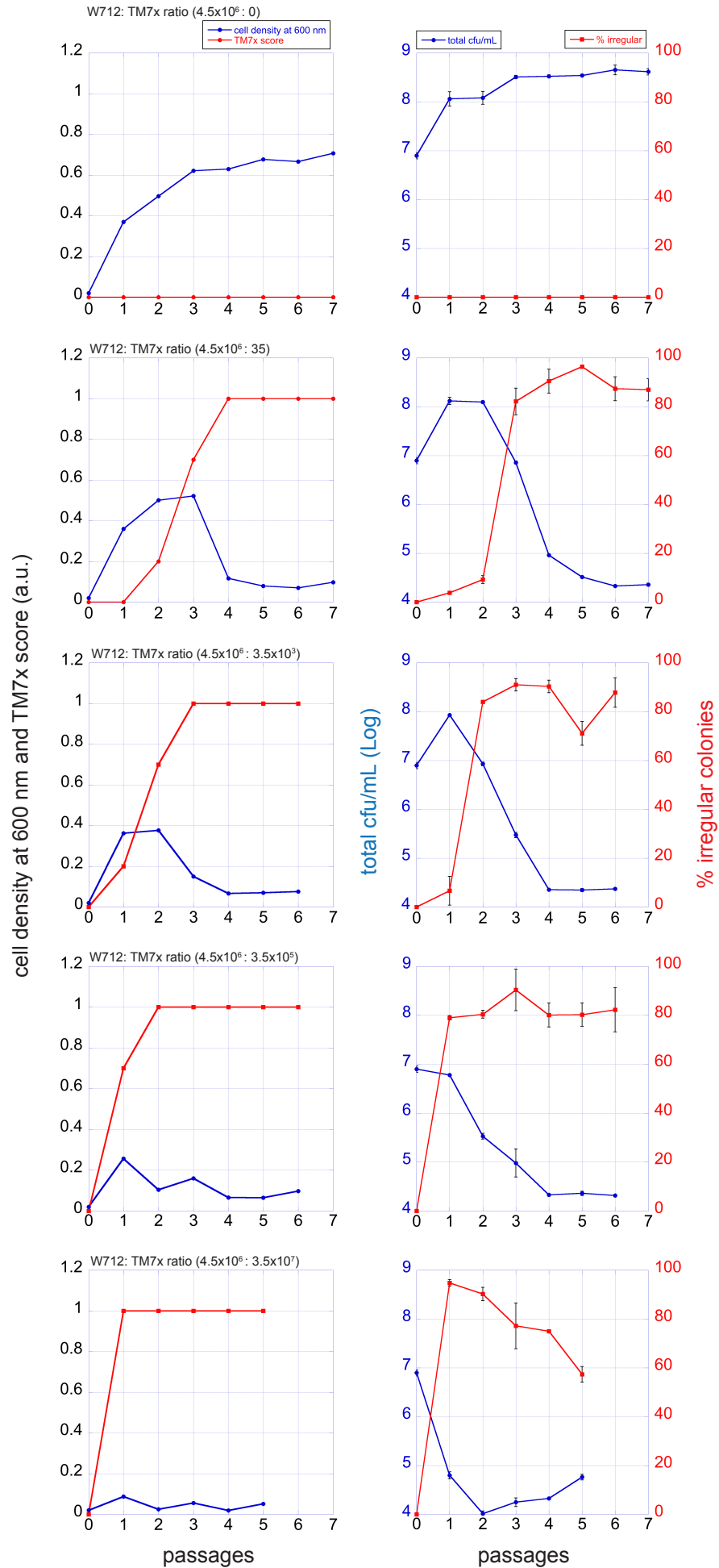

Supplement: Supplementary file 6 — Figure S5 [file 41396_2020_736_MOESM6_ESM.pdf]

Figure S6

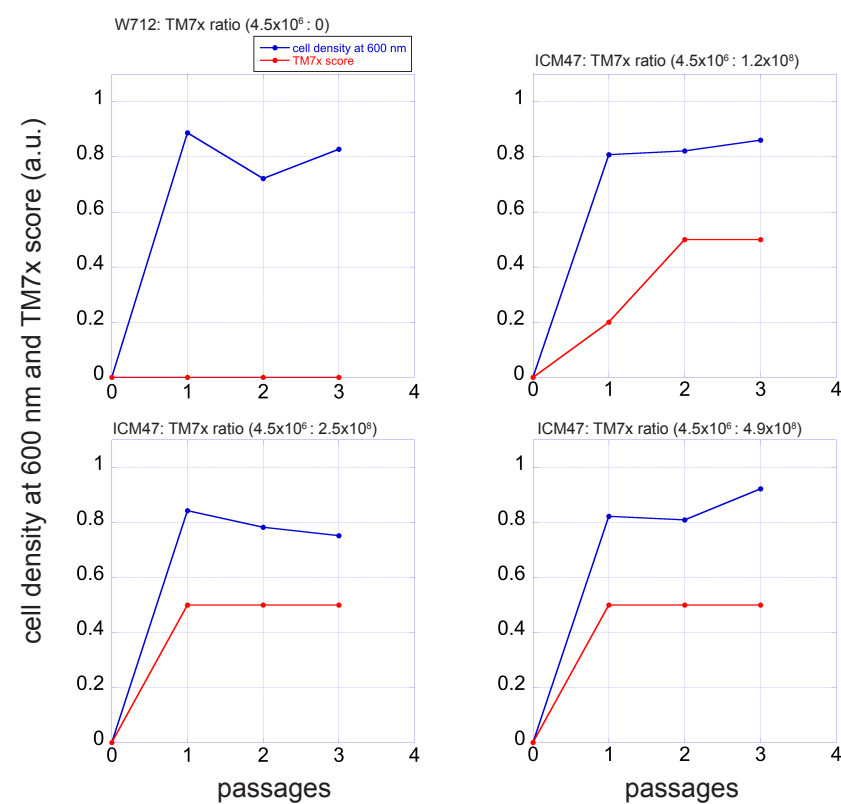

Supplement: Supplementary file 7 — Figure S6 [file 41396_2020_736_MOESM7_ESM.pdf]

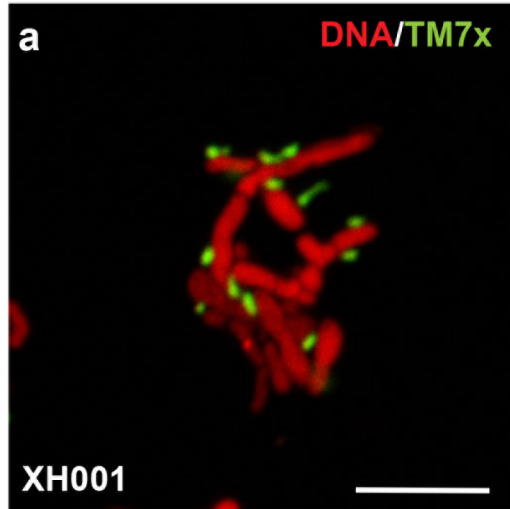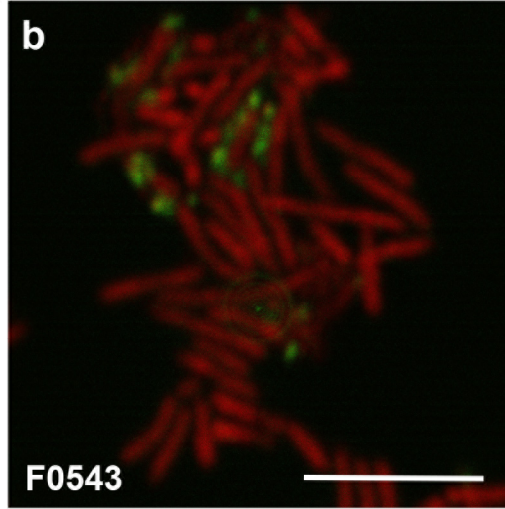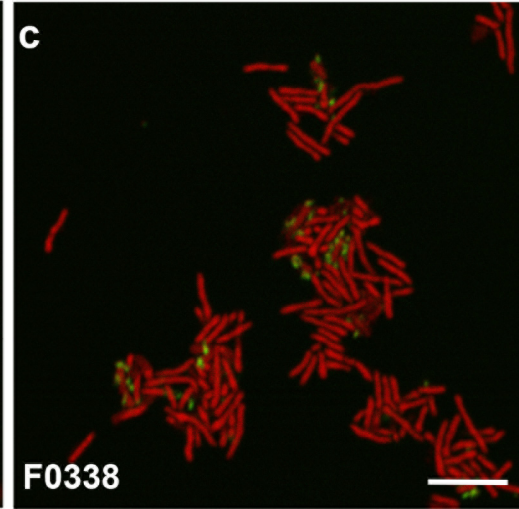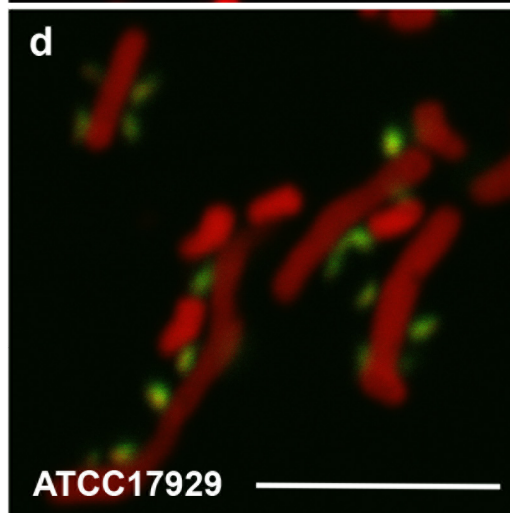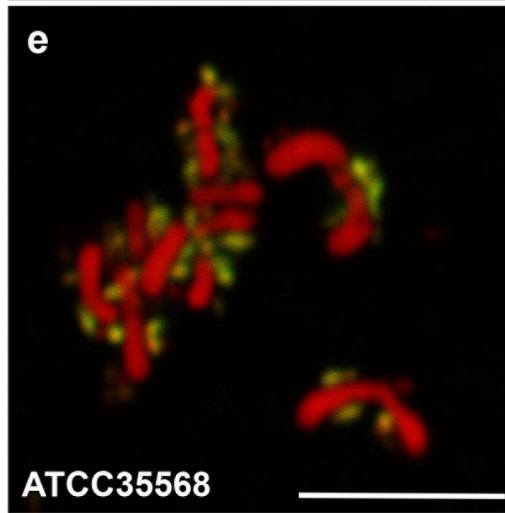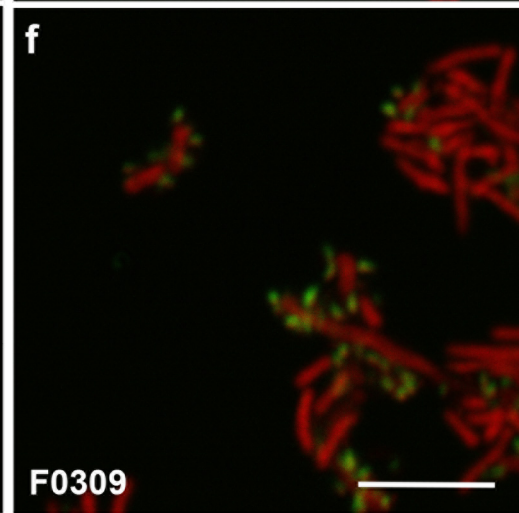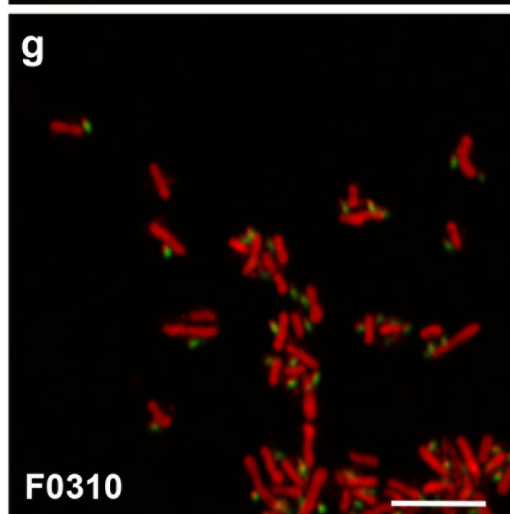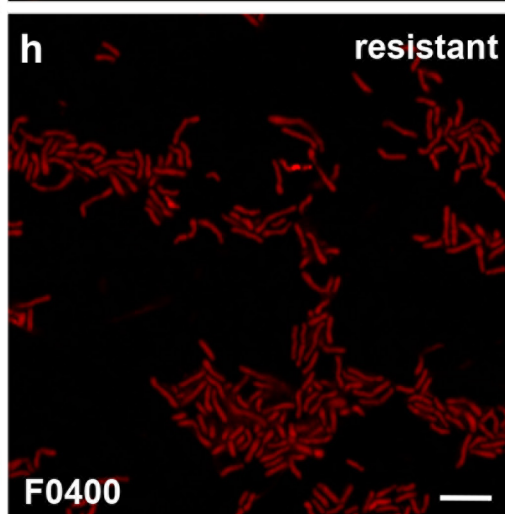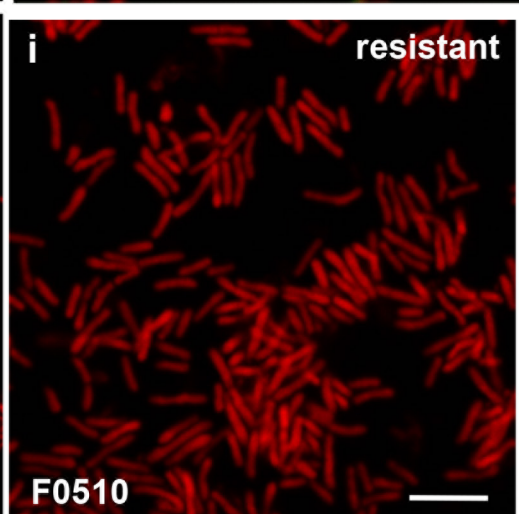

Supplement: Supplementary file 8 — Figure S7 [file 41396_2020_736_MOESM8_ESM.pdf]

a)

Genome

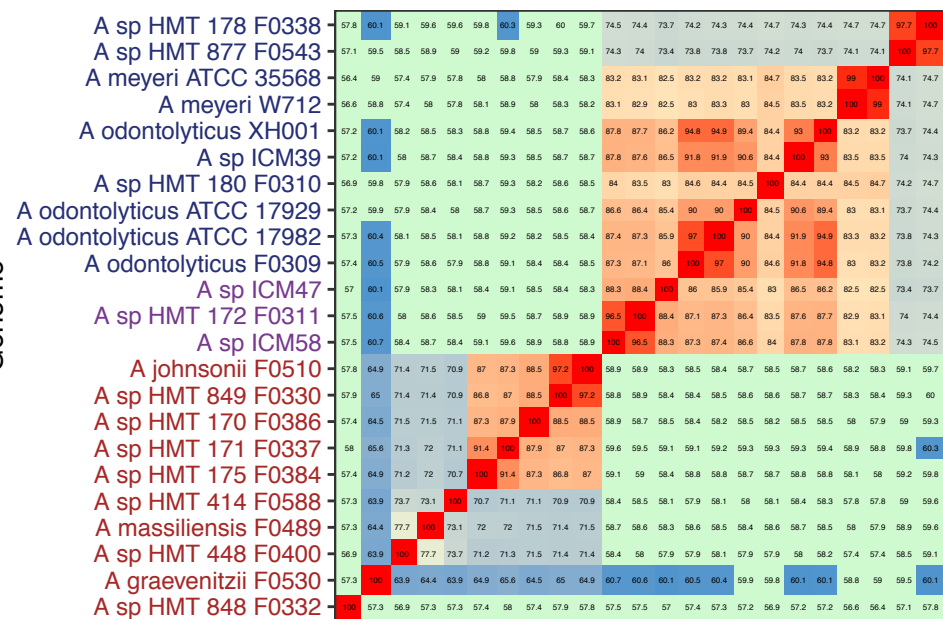

Genome

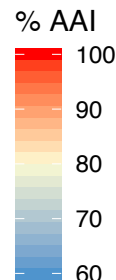

b)

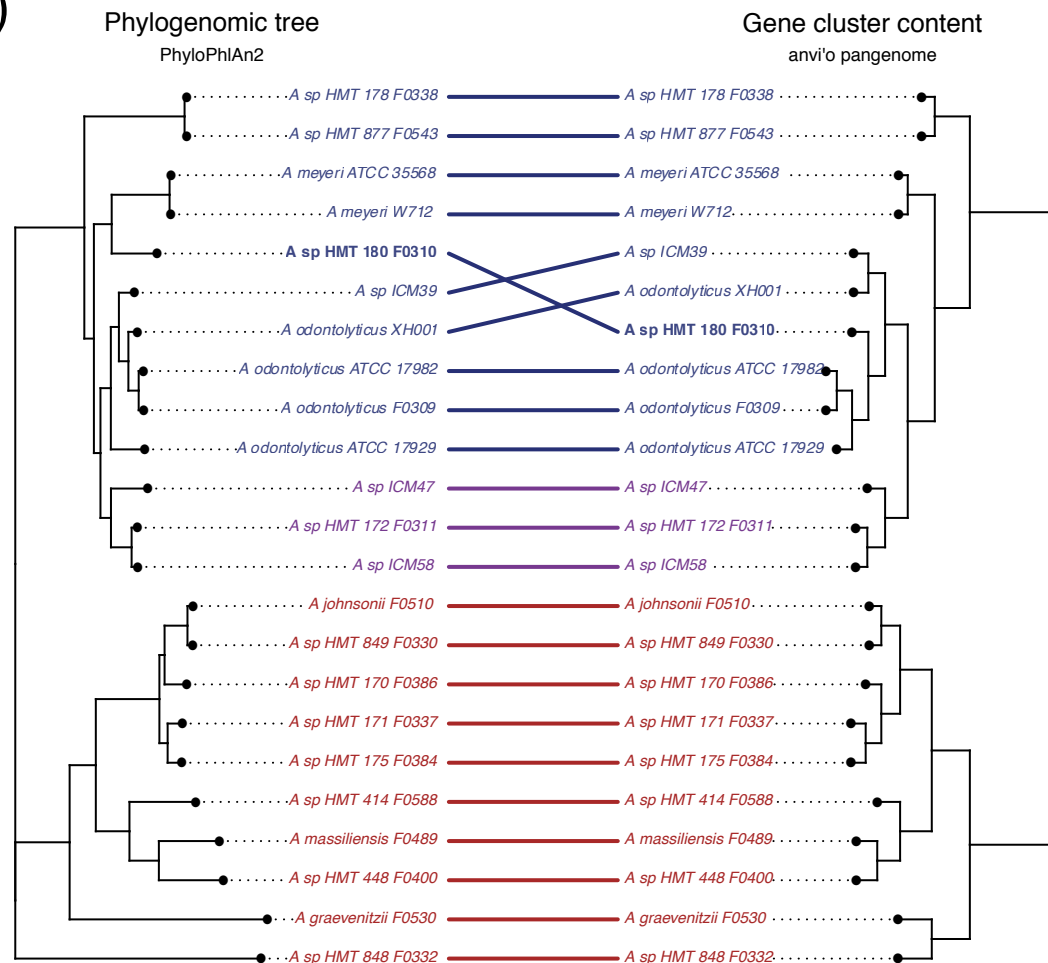

Supplement: Supplementary file 9 — Figure S8 [file 41396_2020_736_MOESM9_ESM.pdf]
